# Knowledge, practices and perceptions of trachoma and its control among communities of Narok County, Kenya

**DOI:** 10.1186/s40794-016-0029-6

**Published:** 2016-07-26

**Authors:** Doris W. Njomo, Jefitha Karimurio, Gladys O. Odhiambo, Mukiri Mukuria, Ernest B. Wanyama, Hillary K. Rono, Micheal Gichangi

**Affiliations:** 1 Kenya Medical Research Institute, P.O. Box 54840-00200, Nairobi, Kenya; 2 Department of Ophthalmology, School of Medicine, College of Health Sciences, University of Nairobi, Kenyatta National Hospital, P.O Box 2683-00202, Off Ngong Road, Old Mbagathi Road, Nairobi, Kenya; 3 Ministry of Health, P.O Box 43319-00100, Nairobi, Kenya

**Keywords:** Trachoma, Knowledge, Practices, Perceptions, Antibiotic treatment, Facial cleanliness, Environmental sanitation

## Abstract

**Background:**

Trachoma is the leading infectious cause of blindness in the world. It is commonly found in cultural groups with poor hygiene. Trachoma control includes **S**urgery, **A**ntibiotics, **F**acial cleanliness and **E**nvironmental Improvement (SAFE). Potentially blinding and active trachoma are monitored using trachomatous trichiasis (TT) in adults and trachoma inflammation-follicular (TF) in children aged 1–9 years respectively. A cross-sectional study to assess the knowledge, practices and perceptions of trachoma and its control was conducted in the endemic communities in Narok County.

**Methods:**

Qualitative methods were used for data collection. Using purposive sampling, 12 focus group discussions (FGDs) with single sex adult and young men and women groups of homogenous characteristics, 12 key informant interviews with opinion leaders and 5 in-depth interviews (IDIs) with trichiasis patients and 6 with persons who have undergone trichiasis surgery were conducted. Data was audio recorded, transcribed, coded and analyzed manually by study themes; knowledge, practices and perceptions of trachoma transmission, infection signs, prevention and control.

**Results:**

Majority of the community members had knowledge of trachoma and its transmission. The practices that contributed to transmission of infection included: failure to wash faces and bathe regularly, sharing of water basins and towels for face washing, traditional methods of trachoma treatment and dirty household environment. Due to socio-cultural perceptions, toilets were unacceptable and use of bushes for human waste disposal was common. Poor perceptions on disease susceptibility, flies on children’s faces, latrine ownership and usage and separation of human and animal dwellings also played a role in the transmission of trachoma. Fear of loss of sight during surgery was a deterrent to its uptake and a desire to be able to see and take care of domestic animals promoted surgery uptake. Majority of the community members were appreciative of Mass Drug Administration (MDA) though side effect such as vomiting and diarrhoea were reported.

**Conclusion:**

Poor practices and related socio-cultural perceptions are important risk factors in sustaining trachoma infection and transmission. Community members require health education for behavior change and awareness creation about surgery, MDA and its potential side effects for elimination of trachoma in Narok County, Kenya.

**Trial registration:**

KEMRI SSC 2785. Registered 2 September 2014.

## Background

**Fig. 1:**
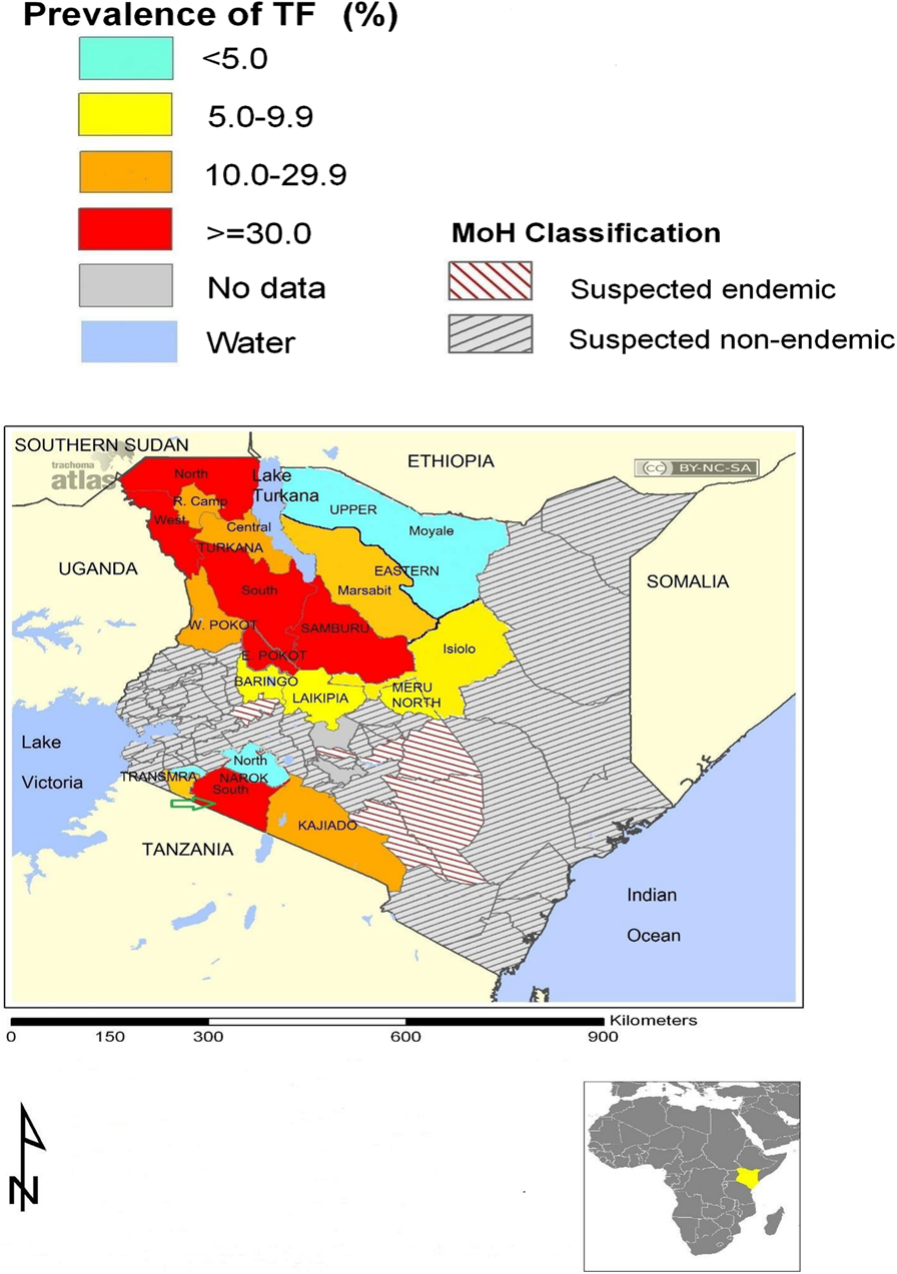
Distribution of active trachoma (TF) in Kenya and Narok (arrow) in 2010

Trachoma is an ancient Neglected Tropical Disease (NTD) which to date is the leading infectious cause of blindness in the world and is caused by the bacterium *Chlamydia trachomatis* [1]. It is usually found in communities with poor hygiene and disappears spontaneously with improving socio-economic status of a community [2]. Lifestyle and culture are known to influence occurrence of trachoma and other eye diseases [3–6]. The distribution of TF in Kenya in 2010 is shown in Fig. 1.

The World Health Organisation (WHO) recommends **S**urgery, **A**ntibiotics, **F**acial cleanliness and **E**nvironmental Improvement (SAFE) for trachoma control. The clinical signs are classified into a simplified WHO trachoma grading scheme [7] as follows: TF = trachoma inflammation-follicular, TI = trachoma inflammation-intense, TS = trachomatous scarring, TT = trachomatous trichiasis and CO = corneal opacity. Prevalence of TT in adults is used as the indicator for the S component of SAFE and prevalence of TF in children aged 1–9 years the indicator AFE components [8–10]. Laboratory testing to verify the prevalence of infection is rarely done because it is considered to be expensive [11].

Community based lid surgery is conducted to prevent blindness in persons with TT, the potentially blinding stage of trachoma. Mass drug administration (MDA) is conducted in communities where prevalence of TF is 10 % or higher. The whole population is treated with antibiotics annually [10] and treatment coverage of at least 80 % is considered successful [9]. Promotion of facial cleanliness and environmental sanitation is conducted in all areas with TF [10].

Narok is one of the trachoma-endemic Counties in Kenya [12] and the disease is mainly found among the nomadic Maasai communities in Narok South [3, 13]. The purpose of this study was to assess the knowledge, practices and perceptions of trachoma and its control and was conducted in the endemic communities in Narok (Fig. 1).

## Methods

### Study site

Narok County is located at the southern-western part of the Rift Valley. The County was created in 2010 when the previous Narok and Trans Mara districts (Fig. 1) were merged. This study was conducted in Narok district. A baseline trachoma prevalence survey was conducted in the district in 2004 (13) The entire district was trachoma endemic and implementation of the full SAFE strategy commenced in 2008. Impact surveys were conducted in 2010 (14) and 2014. In 2010, Narok district was divided into 5 segments and SAFE strategy continued in 2 remaining endemic South Eastern and South Western Narok segments (Fig. 1). The other 3 segments (North Eastern, North Western and Central) were excluded from further MDA since the prevalence of TF was less than 5 % (Fig. 1). A segment was defined as a geographical area with 100,000 – 200,000 persons in line with the WHO recommended “trachoma district” with approximately 100,000 people (10). In 2014 there was no significant change in the prevalence of TF in the 2 southern segments. This status quo was attributed to inadequate FE interventions (14). The results of the 2014 study have been submitted elsewhere for publication.

### Study design and area

The study design was cross-sectional and involved qualitative methods (focus group discussions and in-depth interviews) for data collection. It was conducted in the two endemic segments identified during the 2010 impact assessment survey [13]. A segment is a geographical area with 100,000 to 200,000 persons which correspond to the WHO recommended “trachoma district” [10]. The segments were South Eastern and South Western Narok, (Fig. 1). The other three segments (Central, North Eastern and North Western) were excluded from MDA because they had achieved a TF prevalence of <5 %, the elimination threshold for TF as a public health problem (10).

### Study population

Focus Group Discussions (FGDs) were conducted with community members to gauge community knowledge, practices and perceptions towards trachoma control. Key Informant Interviews (KIIs) were conducted with opinion leaders on trachoma causes, prevention, community members’ practices and perceptions. In addition, separate in-depth interviews (IDIs) were conducted with persons having trichiasis and those who had undergone trichiasis surgery to assess their awareness and perceptions of uptake of surgery. The KIIs were conducted before the FGDs and IDIs in order to get views of opinion leaders about trachoma to facilitate focused questions to understand the feelings of the general community members, thus all sources of data were used to complement one other. A minimum of 4 FGDs is considered adequate for a given research question with a given group of target study participants [14], and a total of 12 FGDs were carried out with adult and youth male and female single-sex groups of homogenous characteristics by trained moderators and note-takers using *Kimasaai*, the local language. The Groups were drawn from Osupuko (4), Ololung’a (5), Mau (2) and Mara (1) administrative divisions in Narok. Twelve (12) KIIs were conducted among the opinion leaders (8 male and 4 female). Four of the participants were from Osupuko, 3 from Mara, 3 from Ololung’a and 2 from Mau Division. A total of 5 in-depth interviews (4 female and one male) were conducted among people with trichiasis (TIDI: **T**richiasis **I**n-**d**epth **I**nterview) all from Mara Division. Another six (6) in-depth interviews (4 female and two male) were conducted among people who had undergone surgery for trichiasis (SIDI: **S**urgery **I**n-**d**epth **I**nterview). Three (3) of the participants were from Osupuko, 2 from Mara and one from Naroosura Division. All the respondents were purposively selected [15]. Standard procedures were observed during data collection [16]. Notes were taken during data collection and audiocassettes used to tape record all the information in the local language. Permission to tape-record was sought from the study participants. The tapes were later transcribed and translated into English.

### Data management and analysis

The data were typed into MS Word and analysed manually according to the study themes which included: knowledge of trachoma transmission and signs of infection: practices in trachoma treatment, facial cleanliness, household environmental cleanliness and toilet use and ownership: perception of risk, of mass antibiotic administration and tetracycline eye ointment, of surgery uptake, flies, latrine use and ownership and separation of animal and human dwellings. Quantitative data from the socio-demographic profiles was analyzed using excel spreadsheets.

### Background characteristics of the study participants

#### Focus Group Discussions (FGD) participants

The single-sex adult and young male and female FGDs participants included adults (35 years and above), (7 FGDs) and young male and female (18 to 34 years), (5 FGDs) respondents of homogenous characteristics. An FGD contained a minimum of 7 and a maximum of 12 participants.

#### Key Informant Interview (KII) participants

The oldest interviewed key informant was 45 years old and the youngest, 25. Eleven (7 male and the 4 female) were in marital unions while one was single. All the interviewed key informants were Christians (Table 1).

**Table 1:** Background characteristics of the Key Informant Interview (KII) participants

Variable		Male	Female
	Number of participants	8	4
Age	Median [range]	37 [31–45]	29 [25–31]
Marital status	Married	7	4
	Single	1	0
Education	None/incomplete primary	1	3
	Primary	1	2
	Secondary	1	2
	Post-secondary	1	1
Main occupation	Teacher	2	1
	Farmer	2	2
	Community leader	3	1
	Religious leader	1	0

#### Persons with trichiasis and Persons who had undergone surgery for trichiasis

The oldest participant with trichiasis interviewed, was 75 years old male and the youngest, 44 female. Four of the participants were Christians and one was non-practicing (Table 2).

**Table 2:** Background characteristics of persons with trichiasis and persons who had undergone surgery for trichiasis

Variable		Male	Female
Persons with trichiasis	Number of participants	1	4
Age	Median	75	61 [44–65]
Marital status	Married	1	3
	Widowed	0	1
Education	None	1	4
Expertise	Housewives	0	4
	Pastoralist	1	0
Persons who had undergone trichiasis surgery	Number of participants	2	4
Age	Median	73 [66–80]	48 [36–95]
Marital status	Married	2	3
	Widowed	0	1
Education	None	0	3
	Primary school	2	1
Main occupation	Housewives	0	3
	Business	0	1
	Farmers	2	0

Among the participants who had undergone surgery for trichiasis, the oldest to be interviewed was 95 years old and the youngest, 36. All the 6 participants were Christians (Table 2).

## Results

### Knowledge on transmission

A large majority of the participants in all the FGDs had heard about trachoma and were aware that it is an eye disease caused by poor sanitation and hygiene with participants in one-quarter of the FGDs further indicating that trachoma can be passed from one person to another through sharing of bathing water, basins and towels. The community members refer to trachoma as *enkoe* and trichiasis as *isarik* in the local language. In FGD01, Adult men: majority of the participants stated that: *“Yes we have heard it; it is caused by dust and poor sanitation, poor hygiene also by sharing of clothes and basins and by flies.”*

However, in one-quarter of the FGDs a minority of the participants indicated that trachoma is a disease for the *Imolelian* clan who inherit it from their ancestors.

All the 12 opinion leaders were aware that trachoma is an eye disease which causes blindness with eleven indicating that it is a contagious disease which is transmitted by flies and caused by poor hygiene, not washing the face and hands, sharing of water, basins and towels, dirt and dust. Only one respondent, a 38 years old farmer indicated that trachoma is just a disease from God and that it is not contagious.

Only two of the persons with trichiasis were aware that it was a disease caused by dirt and transmitted by flies while the remaining 3 indicated that they had no idea as to what the cause of their problem was. TIDI02, a 65 years old female said that:

*“I really do not know but I think it is caused by smoke and the pimples in my eyes too.”*

Furthermore, two of the persons who had undergone trichiasis surgery indicated that trachoma is caused by smoke and old age with one saying that the disease is inherited while 4 mentioned that they did not know the cause. One respondent further stated that a community health worker had told her that the disease is caused by flies. SIDI02, a 45 years old female stated that:

*“I believe it is because of smoky kitchen we use for cooking our meals. I also believe it is caused by inheriting it from our parents because my mother also had it.”*

With regards to keeping domestic animals outside the household compound so as to prevent transmission, a large majority of the participants in all the FGDs were aware that building for their domestic animals away from their households would help prevent trachoma. In FGD05, young men, majority of the participants agreed that: *“Yes we know it is a disease that affects the eyes, poor sanitation contributes to its transmission, flies that are a result of the animals near the household also contribute to the transmission of the disease.”*

The FGDs participants however indicated that although it is advisable to keep the animals away in order to reduce flies, it is not possible as they have to protect their domestic animals from raiders, wild animals, cold and rain. Majority further mentioned that having the cows closer was convenient as they would be able to milk them early in the morning before the calves suckled. In FGD 09 among young Male, the participants stated that:

*“We cannot keep the animals away from us because we shelter them from cold and rain. We keep them close to our homesteads because of security purposes as there are so many wild animals around. We keep them in the house so that we can milk the cows in the morning.”*

With regard to having knowledge that one could undergo surgery in order to make their eyes comfortable, all 5 respondents in the category of persons with trichiasis, indicated that they were aware that an operation could be done with all indicating that some people who had undergone surgery had lost their eyesight completely. All the 5 respondents indicated that they knew people such as their neighbours who had undergone eye surgery. TIDI01, a 65 years old female thus stated:

*“I heard that people are operated and others get healed while others don’t get back to see.”*

On reasons as to why the respondents in the category of persons who had undergone surgery decided to go for surgery, all the respondents indicated that it was because they realized they were becoming blind and wanted to be able to perform their usual duties like taking care of their domestic animals. SIDI04, 36 years old female stated that:

*“Looking after my animals was a problem and it became hard to know and identify missing animals. Also, during the time of travelling I needed someone who could lead me and protect me against the wild animals and also show me the way.”*

### Knowledge of signs of infection

Majority of the participants in all FGDs were aware of the signs of trachoma such as teary, red eyes with white substances at the corners, itchy eyes with eyelashes turning inward and rubbing on the eyeballs. On prevention of trachoma, majority of the participants in all FGDs stated that observing personal hygiene including washing of the eyes, not sharing towels and bathing water as well as environmental sanitation were key factors.

Furthermore, one-half (*n* = 6) of the key informants were aware of signs of trachoma infection such as teary, itchy, and red eyes with eye lashes rubbing on the eyeballs. KII01, a 31 years old male teacher stated that: *“Signs of trachoma are tears, scrubbing of eye lashes and sunken eyes; it is caused by dirtiness and flies. It is transmitted through sharing of clothes and towels.”*

On how a person with trichiasis looks, majority of the participants in all the FGDs indicated that the person has red, watery, itchy eyes that are uncomfortable in direct light and that the eyelashes of such persons had come out. Majority of the participants in all the FGDs further indicated that a person with trachoma usually experiences a lot of pain. In FGD07, Adult women, one participant thus stated: *“The eyes are always itchy and have lots of pain, there is redness in the eyes and they are very watery, the eyeball changes colour, there is lots of discharge from the eyes.”*

### Practices in trachoma treatment

Regarding how the community members treat trachoma infection, it was only in one FGD where majority of the participants indicated that swallowing of the antibiotics distributed by Community Health Workers (CHWs) was the treatment used for trachoma. Three quarters (*n* = 9) of the opinion leaders further indicated that the community members use modern medicine/antibiotics given annually and also visit the hospital for treatment.

However, in eleven FGDs, a large majority of the participants indicated that community members treat trachoma by use of breast milk, salty water, and blood from the ear lobe of a goat, boiled traditional herbs known as *oleleshwa* and *oseki* leaves and scratching the affected eyes using tobacco. Pulling out of eye lashes using *embutet* (tweezers) as treatment for trachoma was mentioned by a majority of the participants in one-quarter (*n* = 3) of the FGDs. In FGD09 among female youth, one participant indicated that: *“There is a specialist who helps to scratch the eyes with tobacco, we also use breast milk, it relieves a lot, we at times use salt or tea leaves solution, we use leaves and roots from the forest to relieve pain.”*

Furthermore, one-quarter (*n* = 3) of the key informants indicated that the community members treat trachoma by traditional practices such as by use of herbs, salty water and milk of breastfeeding mothers.

### Practices in facial cleansing and personal hygiene

On whether the community members wash their children’s faces, a majority of the participants in one-half of the FGDs indicated that community members wash faces of the children every morning before they go to school but the children have to share water, basin and towels. In FGD04, adult men, participants stated that: “*Yes they wash but have to share water and basin, they also share the towel*.”

The key informants also gave their opinion on the community’s practices with regards to personal hygiene and two-thirds (*n* = 8) indicated that their community members do not observe personal hygiene as they do not take a bath daily and some stay 2 to 3 days and others 5 to 7 days without taking a bath. KII05, a 45 years old male chief stated that: *“Adults take 5 to 7 days before taking a bath.”*

On matters of children’s hygiene, one-half (*n* = 6) of the opinion leaders indicated that children especially during the school holidays do not observe hygiene and rarely take a bath or change their cloths. KII05, a 45 years old male chief stated that: *“Young children take longer without taking a bath during holidays and weekends when they are not going to school.”* Only two key informants mentioned that the community members take a bath daily though water availability can be a hindrance and they have to minimize its use.

### Practices in toilet use and ownership

On community members’ practices in use of toilet, a large majority (11) of the opinion leaders indicated that pit latrines were acceptable in their communities though some people do not use them as they are expensive to construct and there are spacious bushes around their homes. Only one opinion leader mentioned that latrines are not acceptable amongst his community members as people are in fear of being seen going inside as according to their culture it is shameful. A 36 years old male farmer, KII12 thus stated: *“Most of the community members do not want to construct the toilets. This is because many of the people do fear to enter the toilets because they do not want to be seen by people. They say that it is lack of respect.”*

With regard to whether men, women and children share toilets, 5 of the opinion leaders indicated that their community members can share the toilets, and 2 that sharing was acceptable as long as there were separate doors for women and men.

Five of the opinion leaders however stated that sharing of toilets among men, women and children is not acceptable among their communities as it is regarded as a taboo. A 25 years old female teacher KII06 stated that: *“Men, women and children are not allowed to use the same pit latrine because there is a taboo that a father and his female child cannot use the same toilet.”*

In one-third (*n* = 4) of the FGDs, a large majority of the participants further indicated that even though toilets were acceptable, the family members cannot share as it is a taboo for a daughter to share a toilet with her father-in-law. In FGD04, adult men, a participant stated that: *“It is acceptable but the latrine must be sub-divided according to gender.”* In FGD09 with female youth, one participant stated that: *“Yes it is accepted but it is shameful because at times may be you are on your way out and you meet your father-in-law.”*

Furthermore, a large majority of the participants in all the FGDs indicated that pit latrines were acceptable among their community members although in one-quarter (*n* = 3) of the FGDs, majority of the participants indicated that latrines were expensive and they could not afford to construct them as they have to sub-divide by gender and by age.

#### Practices in disposal of children’s feaces

Regarding the community members’ practices in disposal of young children’s feaces, 10 respondents during the KIIs indicated that it is used as food for dogs or thrown away in the bush. Only one opinion leader mentioned that the modern community members throw it in pit latrines. KII04, a 33 year old female, women group leader thus stated: *“There are no pit latrines so people throw young children’s feaces in the bush. It is also food for dogs.”*

### Risk perceptions

On whether there were some people in the community who were more at risk of trachoma infection than others, all the opinion leaders indicated that there were, with two-thirds (*n* = 8) mentioning that trachoma was more common among children and one-half (*n* = 6) that it is more common among the old people because they did not observe personal hygiene. One-third (*n* = 4) of the opinion leaders indicated that women were the most affected as they spent time in smoky unventilated houses.

Only a sixth (*n* = 2) of the opinion leaders indicated that trachoma was more common among the *Imolelian* clan who had inherited from their ancestors. A 38 year old male, farmer, KII08 stated that: “The *Imolelian clan was the real owner of trachoma depending on our traditions*.”

Furthermore, a large majority of the participants in 11 FGDs indicated that children were more at risk of getting trachoma while in 10 FGDs a few participants indicated that it was the old people as they did not observe hygiene. However, in three-quarters (*n* = 9) of the FGDs, a few participants indicated that women were more at risk of getting trachoma because they were always in smoky rooms.

In 3 FGDs, the participants indicated that trachoma is a disease that is inherited and the *Imolelian* clan was more at risk of getting infected. It was only in three-quarters (*n* = 9) of the FGDs that a large majority of the participants indicated that everyone who does not observe personal hygiene is at risk of getting trachoma. In FGD05 with young men, one participant stated that: “*Everyone with poor sanitation, even if it is the youth so long as they do not maintain personal hygiene can get trachoma*.”

### Perception of oral azithromycin and tetracycline eye ointment

On perception of medicine distributed every year for trachoma, a large majority of the participants in 11 FGDs indicated that the medicine is good and it has helped improve the health of the people in general and that the distribution should continue every year. However, in one-half (*n* = 6) of the FGDs majority of the participants indicated that the drugs cause side effects such as vomiting and diarrhoea and requested that awareness creation be done before the distribution exercise which would make more people to take the drugs. It was only in one FGD where a large majority of the participants indicated that the drugs were not good at all. In FGD12 among adult women, one participant stated that: *“The drugs have side effects they are not good at all, they cause vomiting and stomach-ache, change the drugs if you can.”*

Majority (*n* = 11) of the opinion leaders indicated that the drugs were good for their people and that many took them with two-thirds (*n* = 8) indicating that some community members complained of side effects such as vomiting, diarrhoea, dizziness and general malaise. Five of the opinion leaders indicated that there was need to create awareness and give health education to the community members prior to drug administration for the people to be well prepared. Only one opinion leader mentioned that there were no trachoma drugs distributed in his community in 2013. KII02, a 31 years old male businessman thus stated:

*“The medicine given annually helps prevent trachoma, they brought the medicine in 2013 and everybody took. After taking people felt hungry and some vomited. People should be given prior notice so that they can get prepared.”*

Regarding preference of tetracycline eye ointment or oral antibiotics, a large majority of the participants in 7 FGDs indicated that they preferred the oral antibiotics as they would also fight other diseases in the body and one was required to swallow only once in a year. In three-quarters of the FGDs, a majority of the participants however indicated that eye ointment was more preferable as one would put it directly on the eye where the infection was, that the ointment had no side effects and it was good for removing all the dirt from the eyes and helping improve visibility. In FGD07 among adult women, one participant stated that: *“I prefer eye ointment because it is effective, it removes all the dirt in the eye and in the morning your eyes see sharply.”*

Furthermore, seven (7) opinion leaders indicated that their community members preferred the eye ointment as it did not have any side effects and was put directly on the eyes where the infection is. KII05, a 45 years old, male chief indicated that: *“Community members prefer eye ointment than medicine to swallow because they do not make people vomit or feel tired.”*

Four opinion leaders however stated that both eye ointment and antibiotics were good as ointment was for the children and pregnant women and the tablets were for the adults.

### Perceptions of flies on children’s faces

On perceptions of flies that are often on the children’s faces, 7 of the opinion leaders indicated that community members regard flies as blessings from God, signs of rain, indicators of future richness and as friends which do not transmit disease. Only 2 respondents indicated that community members regard the flies as transmitters of disease. KII04, a 33 years old female, women group leader thus stated: *“The community members believe that flies are not bad but they are a blessing. Mothers do not know whether flies transmit diseases to their children.”*

### Perception of water availability

During the KIIs, all the opinion leaders indicated that unavailability of water is a major challenge especially in the dry months of the year with one-half indicating that their community members have to walk for more than 5 kilometres looking for water and spending even up to 9 h in one day. The remaining one-half indicated that their community members spend about 1 h as they have to walk for distances ranging from 1 to 4 kilometres in search of water. A 31 years old male teacher, KII01 stated that: *“The only source of water is rain. In the dry season people move from one place to another where water is available, they travel for 7 to 12 kilometres to the source of water, some use 9 to 10 h travelling to the water source.”*

Regarding the opinion leaders’ perceptions on challenges of promoting better hygiene practices in the community, two-thirds indicated that lack of awareness, of health education were the major challenges while 7 mentioned that unavailability of sufficient water was the major barrier to promoting better hygiene. Poverty as a challenge of promoting better hygiene practices was mentioned by 5 opinion leaders. KII11, a 40 years old male pastor stated that: *“Challenges include cultural practices, lack of water and water storage facilities, poverty and illiteracy.”*

## Discussion

Results of the current study have shown that majority of the community members are aware that trachoma is an eye disease that is transmitted by flies due to poor hygiene and sanitation. However, there are community members that do not associate flies on the face with trachoma infection and in fact regard them as signs of blessings, riches and rainfall. The presence of flies as a risk factor for active trachoma has been previously documented by several studies [17, 18].

The results of the current study have also demonstrated that majority of the community members are aware of signs of trachoma infection such as watery, red itchy eyes and refer to the disease locally as *enkoe*. A different study conducted in Kenya also reported that caretakers of children with trachoma had high awareness of signs of trachoma infection and further related this to the use of the local name [19]. The current study results also indicate that community members are aware that observing personal hygiene including washing of the eyes, not sharing towels and bathing water as well as environmental sanitation are key factors in prevention of trachoma infection. Although the shared use of towels is considered a key risk factor in the transmission of trachoma, a systematic review and meta-analysis [20] reported that there was no significant relationship in sharing towels and increased risk of trachoma.

Results of the current study further show that in spite of the community members being aware that it is beneficial to have their animal pens constructed away from the human dwellings so as to reduce transmission of infection, they still have the pens close by. Community members prefer to have domestic animals near so that they can protect them from wild animals, cattle raiders and also conveniently milk them in the morning. Efforts in health education to bring about behavior change are needed to influence communities’ behaviors and perceptions. A Cochrane review [21] outlined the presence of domestic animals close to human dwellings as an environmental sanitation issue which needs to be addressed if trachoma is to be controlled.

The current study results also point out that although the community members are aware that trachoma is treated by swallowing of antibiotics which are distributed annually by community health workers, they have fear of the side effects. A large majority of the community members use other traditional methods such as boiled traditional herbs, breast milk and salty water as well as plucking out of eyelashes to treat trachoma. Similarly, a study conducted in Guinea Bissau reported use of some traditional remedies such as washing the face in urine; or with a paste made by grinding the leaves, flowers and roots of local trees; and cleaning the eyes with lemon juice or grinding chili peppers into the eye [22]. Traditional remedies are reported to continue being used as communities have local perceptions about the etiology and history of trachoma [23].

On facial cleanliness, the results of the current study indicate that caregivers wash the faces of their children daily, although many community members do not observe personal hygiene as they do not take a bath on daily basis. The association between frequent face washing and reduced transmission of trachoma has been reported in other studies [24, 25]. A large-scale randomized trial conducted in Tanzania with an aim of encouraging face washing showed that children with a clean face were at a lower risk of having severe inflammatory trachoma (TI) [23]. Unavailability of water for facial cleanliness and personal hygiene according to the current study results is a contributing factor to trachoma transmission.

The results of the current study show that community members have to walk for long distances in search of water and rely heavily on rainfall for supply of water. Previous studies in pastoralist communities in Turkana region Kenya [26], Gambia [27] and Northern Tanzania [28] reported large families as a major risk factor in the transmission of trachoma. This was associated with the amount of water available to each family member each day. Long distance to water source, low, inadequate amounts of water used per family to clean, lack of latrines and poor hygiene behaviour and practices were reported to be major contributing factors to high transmission rate of trachoma [28]. Similar results have been reported in Northern Tanzania which indicated that long distance to water source, low and inadequate amounts of water used and poor hygiene practices are major contributing factors to the rate of trachoma transmission [28]. A critical review of the SAFE strategy for the prevention of blinding trachoma concluded that among other factors, increasing water supply and quality, and providing health education would help interrupt transmission of trachoma [29].

On the practice of disposing young children’s feaces, the study results indicate that community members either throw children’s feaces in nearby bushes or leave it in the fields for dogs to eat. Only a minority of the modern community members dispose children’s feaces in the latrines. This is an indication that the environment is contaminated and is thus a good reservoir for breeding flies. Results of the current study further showed that the practice of latrine use maybe limited due to socio-cultural beliefs and perceptions which hinder sharing of toilets by all family members. Daughters-in-law are not comfortable visiting the same latrines with their fathers-in-law and believe that sharing is a taboo indicating that each gender would be required to have their own cubicles. Building of separate cubicles of latrines by gender or age would be costly for such communities and generally most members will prefer to use bushes in the absence of separate cubicles rather than share the latrines. This belief contributes to environmental contamination leading to trachoma infection due to the attraction of flies. A study conducted in Ayod County, Sudan, [27] highlighted the importance of communities adopting the behavior of proper disposal of human waste for reduction of transmission of trachoma. A randomized controlled trial examining latrine use [30] showed that latrine provision and very high usage resulted in a decrease of flies on the face, and an associated reduction in trachoma prevalence. Latrines however will only improve environmental sanitation if they are acceptable and used consistently by a large proportion of the community. World Health Organization recommends that latrine provision should be in accordance with what already exists and is acceptable in the community [31].

On being susceptible to trachoma infection, the results of the current study show that majority of the community members are aware that young children and mothers are most at risk of trachoma infection and attribute the susceptibility to poor hygiene. Some community members however have perception that women who spend time in smoky rooms are more at risk of infection. Cromwella et al. [32] reported a higher risk of trichiasis in women compared with men, thus supporting the opinion that women have a greater burden of trichiasis. Similarly results of a study conducted in the Gambia and Tanzania [23] concluded that the lack of communities to relate childhood active infection and trichiasis-induced adult blindness was a major obstacle to SAFE implementation. Results of the current study have further shown that some community members have a misperception that it is the *Imolelian* clan who had inherited the disease from their ancestors who are more at risk of trachoma. Similarly, rural participants in the study conducted in Guinea Bissau also expressed the belief that eye disease originated from other islands [22].

The study results indicate that Narok community members have poor perceptions about the drugs distributed during MDA due to their side effects such as vomiting, diarrhoea, dizziness and general malaise and point out for a need for awareness creation on potential side effects. The importance of spending more time in training community health workers about the side-effects of azithromycin and how to guide community members who are to receive the drugs has been underscored in a pilot study conducted in Ghana [33]. Moreover, findings of a study on community drug distributors’ motivation in the Lymphatic Filariasis Elimination Programme in Kenya called for a combined effort by health workers, local administration and mass media in creating awareness about the MDA and related potential side effects [34]. On the one hand, the results of the current study showed that a large proportion of the community members prefer using tetracycline eye ointment for trachoma treatment as it is put directly on the eye where the disease occurs while on the other hand the results also indicated that community members prefer swallowing azithromycin as this is done only once annually and are of the perception that the tablets clear all other infections. Generally, there are those community members who have a preference for swallowing antibiotics and there are those who prefer using tetracycline eye ointment for trachoma treatment. Randomized controlled trials have demonstrated that the two treatments are equally efficacious [35] azithromycin being more effective in operational use [36]. Tetracycline is almost universally available but the length of administration and difficulty or unpleasantness in application has poor compliance outcome [30]. Azithromycin on the other hand which is active against extra-ocular C. *trachomatis* is well tolerated by both adults and children and achieves good compliance [35, 37].

The results of the study show that although some community members had undergone successful trichiasis surgery, fear of losing eyesight is a barrier to surgery uptake among those who had not had surgery. Patients with trichiasis did not comply with surgery as they reported to know of persons who had lost their sight after undergoing surgery. A study conducted in Ethiopia recommended that the patients who had had a positive experience of surgery could be good campaigners in motivating other community members with trichiasis to undergo surgery [38].

## Conclusions

Health education and promotion activities for awareness creation with an aim of changing cultural perceptions and practices that contribute to trachoma transmission need to be provided to the communities. Community sensitization during MDA is important so that the communities get to understand the benefits of taking the azithromycin and why tetracycline eye ointment should be an option for young children and expectant mothers. The communities need to be encouraged to build and utilize latrines for human waste disposal and the County Government of Narok should consider providing water to promote proper hygiene practices that will help control trachoma transmission.

### Limitations of the study

This was purely a qualitative study and the purpose was to explore and describe knowledge, practices and perceptions of trachoma infection and control among purposively selected study participants. The results are not generalizable as the sample size of a qualitative study is not representative but can be used to build theory through tentative hypotheses.

## Abbreviations

CO, corneal opacity; FGD, focus group discussion; IDI, In-depth interview; KII, key informant interview; MDA, mass drug administration; NTD, neglected tropical disease; SAFE, surgery, antibiotic, facial cleanliness & environmental sanitation; SIDI, surgery In-depth interview; TF, trachomatous inflammation, follicles; TIDI, trichiasis In-depth interview; TS, trachomatous inflammation, intense; TT, trachomatous trichiasis; WHO, world health organization
